# Prevalence and outcome of dual aortic stenosis and cardiac amyloid pathology in patients referred for transcatheter aortic valve implantation

**DOI:** 10.1093/eurheartj/ehaa170

**Published:** 2020-04-08

**Authors:** Paul R Scully, Kush P Patel, Thomas A Treibel, George D Thornton, Rebecca K Hughes, Sucharitha Chadalavada, Michail Katsoulis, Neil Hartman, Marianna Fontana, Francesca Pugliese, Nikant Sabharwal, James D Newton, Andrew Kelion, Muhiddin Ozkor, Simon Kennon, Michael Mullen, Guy Lloyd, Leon J Menezes, Philip N Hawkins, James C Moon

**Affiliations:** Barts Heart Centre, St Bartholomew’s Hospital, West Smithfield, London EC1A 7BE, UK; Institute of Cardiovascular Science, University College London, Gower Street, London WC1E 6BT, UK; Barts Heart Centre, St Bartholomew’s Hospital, West Smithfield, London EC1A 7BE, UK; Institute of Cardiovascular Science, University College London, Gower Street, London WC1E 6BT, UK; Barts Heart Centre, St Bartholomew’s Hospital, West Smithfield, London EC1A 7BE, UK; Institute of Cardiovascular Science, University College London, Gower Street, London WC1E 6BT, UK; Barts Heart Centre, St Bartholomew’s Hospital, West Smithfield, London EC1A 7BE, UK; Barts Heart Centre, St Bartholomew’s Hospital, West Smithfield, London EC1A 7BE, UK; Institute of Cardiovascular Science, University College London, Gower Street, London WC1E 6BT, UK; Barts Heart Centre, St Bartholomew’s Hospital, West Smithfield, London EC1A 7BE, UK; Institute of Health Informatics, University College London, 222 Euston Road, London NW1 2DA, UK; Nuclear Medicine, Abertawe Bro Morgannwg University Health Board, 4 Seaway Parade, Port Talbot SA12 7BR, UK; National Amyloidosis Centre, University College London, Rowland Hill Street, London NW3 2PF, UK; Barts Heart Centre, St Bartholomew’s Hospital, West Smithfield, London EC1A 7BE, UK; William Harvey Research Institute, Queen Mary University of London, Charterhouse Square, London EC1M 6BQ, UK; John Radcliffe Hospital, Oxford University Hospitals, Headley Way, Headington, Oxford OX3 9DU, UK; John Radcliffe Hospital, Oxford University Hospitals, Headley Way, Headington, Oxford OX3 9DU, UK; John Radcliffe Hospital, Oxford University Hospitals, Headley Way, Headington, Oxford OX3 9DU, UK; Barts Heart Centre, St Bartholomew’s Hospital, West Smithfield, London EC1A 7BE, UK; Barts Heart Centre, St Bartholomew’s Hospital, West Smithfield, London EC1A 7BE, UK; Barts Heart Centre, St Bartholomew’s Hospital, West Smithfield, London EC1A 7BE, UK; Barts Heart Centre, St Bartholomew’s Hospital, West Smithfield, London EC1A 7BE, UK; Institute of Cardiovascular Science, University College London, Gower Street, London WC1E 6BT, UK; William Harvey Research Institute, Queen Mary University of London, Charterhouse Square, London EC1M 6BQ, UK; Barts Heart Centre, St Bartholomew’s Hospital, West Smithfield, London EC1A 7BE, UK; Institute of Nuclear Medicine, University College London, 235 Euston Road, London NW1 2BU, UK; NIHR University College London Hospitals Biomedical Research Centre, 149 Tottenham Court Road, London W1T 7DN, UK; National Amyloidosis Centre, University College London, Rowland Hill Street, London NW3 2PF, UK; Barts Heart Centre, St Bartholomew’s Hospital, West Smithfield, London EC1A 7BE, UK; Institute of Cardiovascular Science, University College London, Gower Street, London WC1E 6BT, UK

**Keywords:** Aortic stenosis, Cardiac amyloidosis, TAVI

## Abstract

**Aims:**

Cardiac amyloidosis is common in elderly patients with aortic stenosis (AS) referred for transcatheter aortic valve implantation (TAVI). We hypothesized that patients with dual aortic stenosis and cardiac amyloid pathology (AS-amyloid) would have different baseline characteristics, periprocedural and mortality outcomes.

**Methods and results:**

Patients aged ≥75 with severe AS referred for TAVI at two sites underwent blinded bone scintigraphy prior to intervention (Perugini Grade 0 negative, 1–3 increasingly positive). Baseline assessment included echocardiography, electrocardiogram (ECG), blood tests, 6-min walk test, and health questionnaire, with periprocedural complications and mortality follow-up. Two hundred patients were recruited (aged 85 ± 5 years, 50% male). AS-amyloid was found in 26 (13%): 8 Grade 1, 18 Grade 2. AS-amyloid patients were older (88 ± 5 vs. 85 ± 5 years, *P* = 0.001), with reduced quality of life (EQ-5D-5L 50 vs. 65, *P* = 0.04). Left ventricular wall thickness was higher (14 mm vs. 13 mm, *P* = 0.02), ECG voltages lower (Sokolow–Lyon 1.9 ± 0.7 vs. 2.5 ± 0.9 mV, *P* = 0.03) with lower voltage/mass ratio (0.017 vs. 0.025 mV/g/m^2^, *P* = 0.03). High-sensitivity troponin T and N-terminal pro-brain natriuretic peptide were higher (41 vs. 21 ng/L, *P* < 0.001; 3702 vs. 1254 ng/L, *P* = 0.001). Gender, comorbidities, 6-min walk distance, AS severity, prevalence of disproportionate hypertrophy, and post-TAVI complication rates (38% vs. 35%, *P* = 0.82) were the same. At a median follow-up of 19 (10–27) months, there was no mortality difference (*P* = 0.71). Transcatheter aortic valve implantation significantly improved outcome in the overall population (*P* < 0.001) and in those with AS-amyloid (*P* = 0.03).

**Conclusions:**

AS-amyloid is common and differs from lone AS. Transcatheter aortic valve implantation significantly improved outcome in AS-amyloid, while periprocedural complications and mortality were similar to lone AS, suggesting that TAVI should not be denied to patients with AS-amyloid.

**See page 2768 for the editorial comment on this article (doi: 10.1093/eurheartj/ehaa458)**

## Introduction

Aortic stenosis (AS) is common, with nearly 5% of patients aged 75 and over having at least moderate AS.^1^ ^,^ ^2^ Symptomatic severe AS requires treatment, either in the form of surgical or transcatheter aortic valve replacement (TAVI), without which average survival is only 2–3 years.^3–7^ Transcatheter aortic valve implantation numbers are increasing worldwide, a trend that is likely to continue due both to the ageing population and the expanding role of the procedure itself to include intermediate^8^ and even low-risk populations.^9^ Cardiac amyloidosis (CA) is also common, particularly in elderly males, with wild-type transthyretin amyloid (ATTR) deposits seen in the heart at autopsy in 25% of patients aged 85 and over.^10^ So, it follows that CA is likely to co-exist in a significant proportion of elderly patients with AS. There is now a non-invasive diagnostic strategy for ATTR-CA, supported by recent expert consensus recommendations^11^ ^,^ ^12^: bone scintigraphy (^99m^Tc-3,3-diphosphono-1,2-propanodicarboxylic acid, DPD; ^99m^Tc-pyrophosphate; or ^99m^Tc-hydroxymethylene diphosphonate) coupled with a negative search for a plasma cell dyscrasia.^11^ ^,^ ^12^ Several studies have shown a prevalence of dual aortic stenosis and cardiac amyloid pathology (AS-amyloid) of 14–16% in elderly TAVI patients,^13^ ^,^ ^14^ but outcome data are not yet available. Transcatheter aortic valve implantation in AS-amyloid may be futile if outcome post-TAVI is no better than medical management.^15^ The most comprehensive paper showed that AS-amyloid was more common in men and was associated with a low-flow, low-gradient AS.^14^ ^,^ ^16^ However, recruited patients were more male than typical TAVI populations, the number referred clinically (i.e. with potential bias) is unclear, and bone scintigraphy was performed pragmatically after TAVI, so periprocedural complications preventing recruitment (e.g. mortality) would not be captured.^14^

By recruiting pre-procedure with blinding of the TAVI team to the bone scintigraphy results, we aimed to confirm the prevalence of AS-amyloid across two sites in the elderly referred for TAVI and identify its differences to lone AS for presentation, periprocedural complications and mortality.

## Methods

This work forms part of ATTRact-AS (*a study investigating the role of occult cardiac amyloid in the elderly with aortic stenosis*, NCT03029026). This study complies with the Declaration of Helsinki, relevant local ethics and site approvals were obtained and all patients provided informed consent. Recruitment took place across two sites: Barts Heart Centre, London (October 2016 to January 2019) and John Radcliffe Hospital, Oxford (January 2018 to June 2019). To reduce bias, recruitment took place when referred for TAVI, but typically before the multidisciplinary team meeting. We therefore anticipated some cross-over to medical therapy and rarely to surgical valve replacement, which occurred. Baseline assessment included blood tests for high-sensitivity troponin T (hsTnT) and N-terminal pro-brain natriuretic peptide (NT-proBNP), a 6-min walk test and EQ-5D-5L health questionnaire in addition to routine clinical care—including electrocardiogram (ECG) and echocardiogram.

### Electrocardiogram

Sokolow–Lyon criteria was calculated as the sum of the voltage of the S-wave in lead V1 and the R-wave in lead V5 or V6 (whichever was greater).^17^ The voltage/mass ratio (V/M ratio) was defined as the Sokolow–Lyon total divided by the indexed left ventricular (LV) mass by echocardiography.^18^ Low limb lead voltages were defined as all limb leads with an amplitude ≤0.5 mV.

### Echocardiography

All patients underwent clinical transthoracic echocardiogram, primarily for assessment of AS severity (including aortic valve peak velocity, mean gradient, and valve area quantification), any concomitant valve pathology, left and right ventricular systolic and left ventricular diastolic, according to the local protocol, which is based on the American Society of Echocardiography, the British Society of Echocardiography, as well as the European Association of Cardiovascular Imaging guidelines.^19–21^ Left ventricular ejection fraction (LVEF) was calculated using Simpson’s biplane whenever possible, or otherwise quantified visually. Stroke volume (SV) was quantified using the left ventricular outflow tract (LVOT) velocity time integral and the LVOT diameter and then indexed to body surface area. Relative wall thickness was defined as: (2 × posterior wall thickness)/(LV internal diameter at end-diastole).^22^ Left ventricular mass was calculated using the formula from Devereux *et al.* ^23^:

$$\text{LV}\;\text{mass}=0.8\;x\;1.04\;x\;\left[{\left(\text{IVSd}+\text{LVIDd}+\text{PWd}\right)}^{3}–{\;\text{LVIDd}}^{3}\right]+0.6$$

Where IVSd is the interventricular septal diameter, LVIDd is the left ventricular internal dimension in diastole, and PWd is the posterior wall diameter, all measured in 2D. Longitudinal strain analysis was performed offline by an accredited echocardiographer using Tomtec software.

Myocardial contraction fraction (MCF), which indexed the SV to the myocardial volume, was calculated as previously described.^24^ Disproportionate hypertrophy was defined as an IVSd greater than 1.9 cm in males or 1.6 cm in females. ‘Classical’ low-flow, low-gradient was defined as an aortic valve area ≤1.0 cm^2^, with an LVEF <50%, an indexed SV <35 mL/m^2^, a peak aortic valve velocity <4 m/s, and a mean gradient <40 mmHg, conversely ‘paradoxical’ low-flow, low-gradient was defined as an LVEF ≥50%, but an indexed SV <35 mL/m^2^, peak velocity <4 m/s, and mean gradient <40 mmHg.^19^ ^,^ ^25^ Where equivocal, AS severity was adjudicated using low-dose dobutamine stress echocardiography, the computed tomography (CT)-derived aortic valve calcium score or by clinical assessment.^19^ ^,^ ^25^ ^,^ ^26^

### DPD scintigraphy

**Figure 1:**
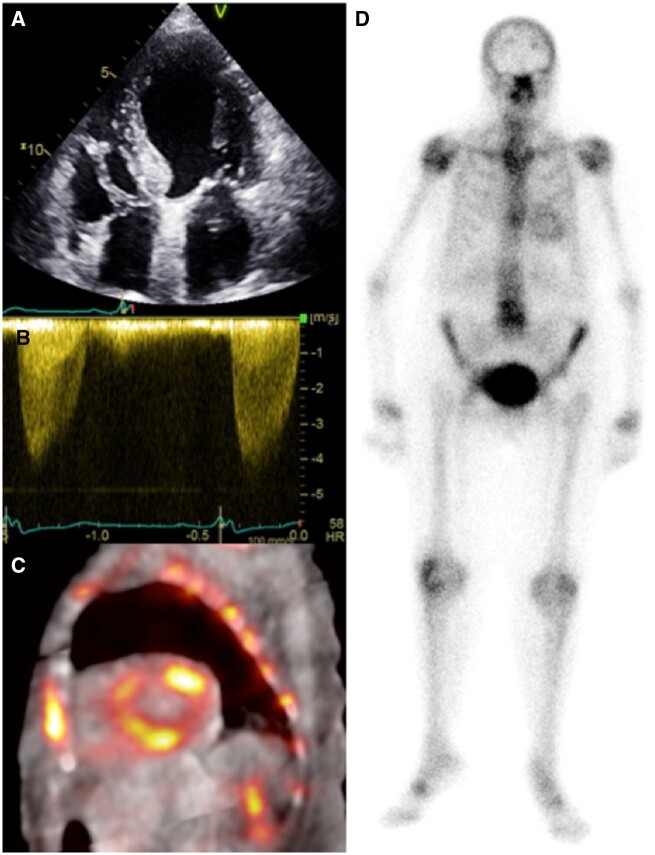
A 90-year-old man with severe aortic stenosis referred for transcatheter aortic valve implantation. Apical four-chamber echocardiography (*A*) shows normal biventricular size with mild left ventricular hypertrophy (1.3 cm basal septum). Continuous-wave Doppler confirmed severe aortic stenosis (peak velocity > 4 m/s) (*B*). The ^99m^Tc-3,3-diphosphono-1,2-propanodicarboxylic acid fused single-photon emission computed tomography/computed tomography short-axis (*C*) confirms diffuse cardiac retention of tracer, visible also on the planar image (*D*), compatible with Perugini grade 2.

All DPD scans were performed using a hybrid Phillips Brightview single-photon emission computed tomography (SPECT)–CT gamma camera, a Siemens Symbia gamma camera or Pulse CDC gamma camera (IS2) following the injection of 700 MBq DPD. The imaging protocol consisted of an optional early (5 min, not performed at the Oxford site) and a late (3 h) planar whole-body image, with a SPECT/CT (or SPECT only) of the chest at 3 h. DPD scans were reported by two experienced clinicians using the Perugini grading system,^27^ where Grade 0 represents no cardiac uptake with normal bone uptake (i.e. negative) and Grades 1–3 represent increasing cardiac uptake with increasing bone attenuation (*Figure 1*). DPD scan findings were also independently reviewed by the National Amyloidosis Centre (NAC). The managing clinicians were blinded to the results of the DPD scan until after the patient’s TAVI procedure (or decision for medical management was made) unless the scan had been requested clinically (*n* = 16, 8%) or there was evidence of a plasma cell dyscrasia on serum free light chains, serum and urine immunofixation (sent when the DPD scan was confirmed positive). All patients with a positive DPD scan were discussed with NAC and, where appropriate, referred for further review. Detailed clinical review for those patients seen at the NAC with a plasma cell dyscrasia included: clinical history and examination, cardiovascular magnetic resonance (where appropriate), serum amyloid P scintigraphy, fat biopsy, genotyping, 6-min walk test, and serum biomarkers.

### Periprocedural complication

Periprocedural complications were defined using the Valve Academic Research Consortium-2 (VARC-2) criteria and occurred *prior to* discharge, except one occurring within 2 months of the procedure.^28^ Mild TAVI aortic valve stenosis was defined using the VARC-2 criteria as a peak velocity ≥3 m/s or a mean gradient ≥20 mmHg and moderate/severe AS was defined as a peak velocity >4 m/s or a mean gradient >40 mmHg.^28^ The degree of post-TAVI aortic regurgitation or AS was based on departmental echocardiography when available (rather than ‘on table’ imaging), which was performed in the majority of patients prior to discharge.

### Follow-up

Follow-up was for mortality using national data via the National Health Service and was 100% complete.

### Statistical analysis

Statistical analysis used IBM SPSS Statistics (Version 25) and Stata/SE (Version 15) software. Where appropriate, results are described as mean ± standard deviation or median ± interquartile range. Normality was initially assessed using the Kolmogorov–Smirnov test and then (when indicated) plotted on a histogram. Kruskal–Wallis one-way analysis of variance was used when comparing more than two groups as the omnibus test, with the Dunn–Bonferroni test for pairwise comparison. The Student’s *t*-test or Mann–Whitney *U* test was used to compare continuous variables and either χ^2^ or Fisher’s exact testing for categorical data. Univariate and multivariable analysis was performed using binary logistic regression with the presence of AS-amyloid as the dependent variable. A two-sided *P*-value <0.05 was considered significant.

## Results

Two hundred patients (mean age 85 ± 5 years, 50% male) were recruited. Comorbidities (hypertension, hypercholesterolaemia, diabetes, pre-intervention atrial fibrillation, and permanent pacemaker) were common (*Table 1*). Baseline LVEF was 54 ± 11%, aortic valve measurements were: peak velocity 4.13 ± 0.65 m/s, mean gradient 41 ± 14 mmHg, area 0.73 ± 0.22 cm^2^. One hundred and forty-nine (75%) patients had undergone TAVI, 2 (1%) surgical aortic valve replacement, and 49 (24%) were being managed medically (15 were for conservative management; the remainder for ongoing surveillance).

**Table 1:** Basic demographics, echo parameters, aortic valve calcium score and clinical parameters for patients with lone AS and those with dual pathology (AS-amyloid) prior to TAVI

	Overall (*n* = 200)	AS-amyloid (*n* = 26)	Lone AS (*n* = 174)	*P*-value
Demographics				
Male	99 (50%)	16 (62%)	83 (48%)	0.19
Age	85 ± 5	88 ± 5	85 ± 5	**0.001**
Clinical parameters				
Hypertension	154 (77%)	19 (73%)	135 (78%)	0.61
Hypercholesterolaemia	84 (42%)	9 (35%)	75 (43%)	0.41
Diabetes mellitus	48 (24%)	3 (12%)	45 (26%)	0.11
Atrial fibrillation	74 (37%)	11 (42%)	63 (36%)	0.55
Permanent pacemaker	23 (12%)	4 (15%)	19 (11%)	0.51
6-MWT (m)	123 (60–260)	94 (48–239)	138 (66–260)	0.36
Pre-TAVI QoL total	60 (50–75)	50 (40–69)	65 (50–75)	**0.04**
ECG parameters				
Heart rate (b.p.m.)	73 ± 16	70 ± 13	73 ± 16	0.32
Low-voltage limb leads	5 (3%)	0	5 (3%)	1.00
S-L criteria (mV)	2.5 ± 0.9	1.9 ± 0.7	2.5 ± 0.9	**0.03**
1st degree AV block^a^	35 (20%)	1 (5%)	34 (22%)	0.08
QRS duration (ms)	110 ± 28	118 ± 27	109 ± 28	0.16
LBBB^a^	20 (11%)	1 (5%)	19 (12%)	0.48
RBBB^a^	28 (16%)	8 (36%)	20 (13%)	**0.01**
Echo parameters				
Left ventricle				
LVEF (%)	54 ± 11	54 ± 14	54 ± 11	0.99
Indexed SV (mL/m^2^)	38 ± 11	34 ± 10	38 ± 11	0.12
IVS diameter (cm)	1.3 ± 0.3	1.4 ± 0.3	1.3 ± 0.2	**0.02**
Posterior wall (cm)	1.1 ± 0.3	1.3 ± 0.4	1.1 ± 0.2	**0.001**
DPH	9 (5%)	2 (8%)	7 (4%)	0.33
RWT (cm)	0.51 ± 0.19	0.61 ± 0.28	0.50 ± 0.16	0.07
Indexed LV mass (g/m^2^)	121 ± 38	136 ± 36	118 ± 38	**0.03**
MCF (%)	23.3 ± 8.9	20.3 ± 8.8	23.8 ± 8.8	0.08
Mitral annular *S*′ (m/s)	0.06 ± 0.02	0.05 ± 0.01	0.06 ± 0.02	**0.04**
GLS (%)	−14 ± 6	−15 ± 6	−14 ± 6	0.29
Diastolic function				
*E*/*A* ratio	0.81 (0.69–1.32)	1.28 (0.75–2.15)	0.78 (0.68–1.22)	0.09
Lateral *E*/*E*′	17 ± 9	20 ± 13	16 ± 8	0.16
MV dec time (ms)	238 ± 86	241 ± 88	237 ± 86	0.84
LA diameter (cm)	4.2 ± 0.7	4.4 ± 0.6	4.1 ± 0.7	0.06
RV function				
TAPSE (cm)	1.9 ± 0.5	1.8 ± 0.5	1.9 ± 0.5	0.56
Aortic valve				
Peak velocity (m/s)	4.12 ± 0.64	3.95 ± 0.73	4.15 ± 0.62	0.15
Mean gradient (mmHg)	41 ± 14	37 ± 14	42 ± 14	0.12
AVA (cm^2^)	0.73 ± 0.22	0.74 ± 0.23	0.73 ± 0.22	0.69
Classical LFLG AS	19 (10%)	3 (12%)	16 (9%)	0.72
Paradoxical LFLG AS	31 (16%)	5 (19%)	26 (15%)	0.57
Composite parameters				
V/M ratio (mV/g/m^2^)	0.024 ± 0.012	0.017 ± 0.007	0.025 ± 0.012	**0.03**
CT parameters				
AV calcium score (HU)	2688 ± 1647	3149 ± 1916	2619 ± 1600	0.17
Blood results				
Creatinine (mmol/L)	107 ± 45	118 ± 38	105 ± 46	0.20
eGFR (mL/min/1.73 m^2^)	55 ± 16	50 ± 14	56 ± 17	0.08
hsTnT (ng/L)	23 (15–37)	41 (25–84)	21 (14–34)	**<0.001**
NT-proBNP (ng/L)	1467 (640–3337)	3702 (1286–5626)	1254 (598–2769)	**0.001**

Values are represented as mean ± standard deviation or median (interquartile range) where appropriate. *P*-values <0.05 are shown in bold.

6-MWT, 6-min walk test; AS, aortic stenosis; AV, aortic valve; AVA, aortic valve area; DPH, disproportionate hypertrophy; eGFR, estimated glomerular filtration rate; GLS, global longitudinal strain; hsTnT, high-sensitivity troponin T; LBBB, left bundle branch block; LV, left ventricle; LVEF, left ventricular ejection fraction; MCF, myocardial contraction fraction; MV dec time, mitral valve deceleration time; NT-proBNP, N-terminal pro-brain natriuretic peptide; QoL, quality of life; RBBB, right bundle branch block; RWT, relative wall thickness; S-L, Sokolow–Lyon criteria; TAPSE, tricuspid annular plane systolic excursion; V/M, voltage/mass ratio.

a Missing ECG data in 4 lone AS and 19 AS-amyloid patients—percentages and statistics quoted reflect this.

### AS-amyloid prevalence and type

AS-amyloid was found in 26 patients, a prevalence of 13% (95% confidence interval 9–18%). The amyloid burden was skewed with no patients with a DPD Perugini Grade 3, but more with Grade 2 than Grade 1 (Grade 1 in 8, Grade 2 in 18, and Grade 3 in 0). A plasma cell dyscrasia was detected in 6 (23%), necessitating unblinding and a referral to the National Amyloidosis Centre, but following detailed review (as described in the methods), AL amyloid was felt unlikely in all cases. All genotyped patients to date (*n* = 12) were wild-type ATTR.

### AS-amyloid vs. lone AS baseline

AS-amyloid patients were 3 years older (88.1 ± 5 vs. 84.7 ± 5 years, *P* = 0.001) and slightly more male, but this did not reach clinical significance (62% vs. 48% male, *P* = 0.19). They had a reduced quality of life at baseline (EQ-5D-5L 50 vs. 65, *P* = 0.04), but the same 6-min walk distance (94 vs. 138 m, *P* = 0.36). There was no difference in comorbidities (*Table 1*). Both had the same aortic valve severity (peak velocity, mean gradient, and valve area) and the same degree of valve calcification by CT.

AS-amyloid had lower Sokolow–Lyon criteria (1.9 ± 0.7 vs. 2.5 ± 0.9 mV, *P* = 0.03) and more right bundle branch block (RBBB) (36% vs. 13%), but low limb lead voltages hardly occurred (*n* = 5 total, all lone AS).

**Figure 2:**
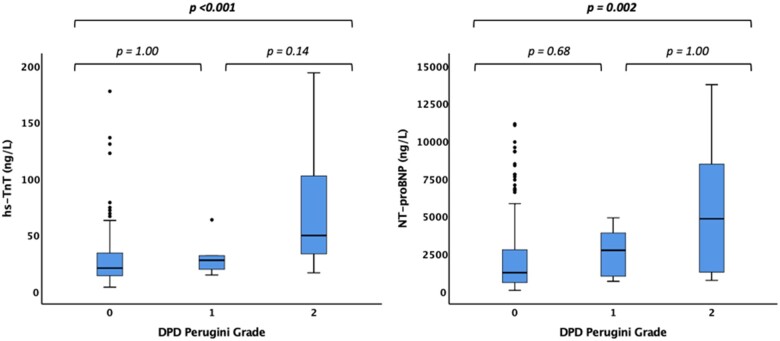
Box and whisker plots demonstrating elevated high-sensitivity troponin T (*A*) and N-terminal pro-brain natriuretic peptide (*B*) in AS-amyloid compared to lone aortic stenosis and with amyloid burden measured by ^99m^Tc-3,3-diphosphono-1,2-propanodicarboxylic acid Perugini grade. Outliers with an high-sensitivity troponin T greater than 200 ng/L and an N-terminal pro-brain natriuretic peptide greater than 150 000 have been excluded from this figure, but included in the statistical analysis (*n* = 2 and *n* = 4, respectively). HsTnT, high-sensitivity troponin T; NT-proBNP, N-terminal pro-brain natriuretic peptide.

When clinically identified, cardiac amyloidosis typically has marked echocardiographic changes. Here, with discovery by DPD scan and in the presence of severe AS and confounding comorbidities, differences were modest: wall thickness was 1–2 mm thicker with higher mass, but no difference in disproportionate hypertrophy (*P* = 0.33). There was no difference in the prevalence of either ‘classical’ (impaired LVEF) low-flow, low-gradient AS (12% vs. 9%, *P* = 0.72) or ‘paradoxical’ (normal LVEF) low-flow, low-gradient AS (19% vs. 15%, *P* = 0.57). Mitral annular *S*′ was slightly lower, but LVEF, SVs, global longitudinal strain, MCF, left atrial size, right ventricular function, and all other diastolic measures were the same. Combining ECG and echocardiography, the V/M ratio was lower (0.017 ± 0.007 vs. 0.025 ± 0.012, *P* = 0.03). N-terminal pro-brain natriuretic peptide and hsTnT were approximately double: hsTnT 41 (25–84) ng/L vs. 21 (14–34) ng/L*, P* < 0.001 and NT-proBNP 3702 (1286–5626) ng/L vs. 1254 (598–2769) ng/L, *P* = 0.001, differences that were not related to renal function. Both hsTnT and NT-proBNP had a dose–response curve with amyloid burden by DPD Perugini grade (*P* < 0.001 and 0.002, respectively for trends, *Figure 2*).

Using multivariable analysis, increasing age, troponin, and the presence of RBBB were associated with AS-amyloid (*Table 2*). Voltage/mass (V/M) ratio was also associated (*P* = 0.045); however, the analysis was performed separately (with age and gender) to avoid underpowering the main model, as the voltage component of the V/M ratio cannot be assessed in bundle branch block or ventricular paced rhythm, present in 30% (Supplementary material online, *Table S3*).

**Table 2:** Univariate and multivariable binary logistic regression analysis, showing that age, hsTnT and the presence of RBBB were all associated with the presence of AS-amyloid

	Univariate analysis		Multivariable analysis		
Variables	*P*-value	Exp (B)	*P*-value	Exp (B)	CI for Exp (B)
6-min walk (per m decrease)	0.48	1.00			
Age (per year increase)	**0.002**	1.15	**0.04**	1.13	1.00–1.27
AVA (per cm^2^ increase)	0.69	2.06			
AV mean PG (per mmHg decrease)	0.12	0.97			
AV *V*_max_ (per m/s decrease)	0.15	0.61			
AV calcium score (per HU increase)	0.18	1.00			
Baseline QoL (per unit decrease)	0.08	0.98			
*E*/*A* ratio (per unit increase)	**0.03**	1.67			
Gender (male)	0.19	1.75			
GLS (%)	0.29	0.96			
hsTnT (per ng/L increase)	**0.001**	1.02	**0.03**	1.02	1.00–1.03
Indexed LV mass (per g/m^2^ increase)	**0.03**	1.01			
Indexed SV (per mL/m^2^ decrease)	0.12	0.97			
IVSd (per cm increase)	**0.02**	6.66			
LA diameter (per cm increase)	0.07	1.90			
Lateral *E*/*E*′ (per unit increase)	**0.04**	1.05	0.56	1.02	0.96–1.08
LBBB	0.29	0.33			
LVEF (per % decrease)	0.99	1.00			
MCF (per % decrease)	0.08	0.95			
Mitral annulus *S*′ (per m/s decrease)	**0.046**	0.00	0.13	0.00	0.00–43 640.00
MV dec time (per ms increase)	0.84	1.00			
NT-proBNP (per ng/L increase)	0.06	1.00	0.41	1.00	1.00–1.00
PWd (per cm increase)	**0.003**	8.72	0.14	3.95	0.65–23.91
RBBB	**0.01**	3.42	**0.005**	6.88	1.81–26.21
RWT (per cm increase)	**0.01**	12.1			
S-L criteria (per mV decrease)	**0.03**	0.39			
TAPSE (per cm decrease)	0.56	0.76			
V/M ratio (per mV/g/m^2^ decrease)	**0.03**	0.00			

S-L criteria and *E*/*A* ratio were not included in the multivariable analysis as S-L criteria cannot be calculated with bundle branch block or paced rhythm (30% of all patients) and *E*/*A* ratio when AF is present (37% of patients). Only one parameter reflecting LV mass was used in the multivariable analysis to avoid confounding. *P*-values <0.05 are shown in bold.

AV, aortic valve; AVA, aortic valve area; CI, 95% confidence interval; Exp (B), exponentiation of the B coefficient; GLS, global longitudinal strain; hsTnT, high-sensitivity troponin T; HU, Hounsfield units; IVSd, interventricular septum diameter; LV, left ventricle; LBBB, left bundle branch block; LVEF, left ventricular ejection fraction; MCF, myocardial contraction fraction; MV, mitral valve; NT-proBNP, N-terminal pro-brain natriuretic peptide; PWD, posterior wall diameter; RBBB, right bundle branch block; RWT, relative wall thickness; S-L, Sokolow–Lyon; TAPSE, tricuspid annular plane systolic excursion; V/M, voltage/mass ratio.

### Periprocedural complications

In the 149 (75%) patients who underwent TAVI (16 AS-amyloid, 133 lone AS), complication rates were equivalent to published international trials.^8^ ^,^ ^15^ Using strict criteria and capturing all major and minor events via VARC-2, complications occurred at the same rate in AS-amyloid, *P* = 0.77 (Supplementary material online, *Table S4*). No patient died within the first 30 days post-TAVI.

### Mortality

**Figure 3:**
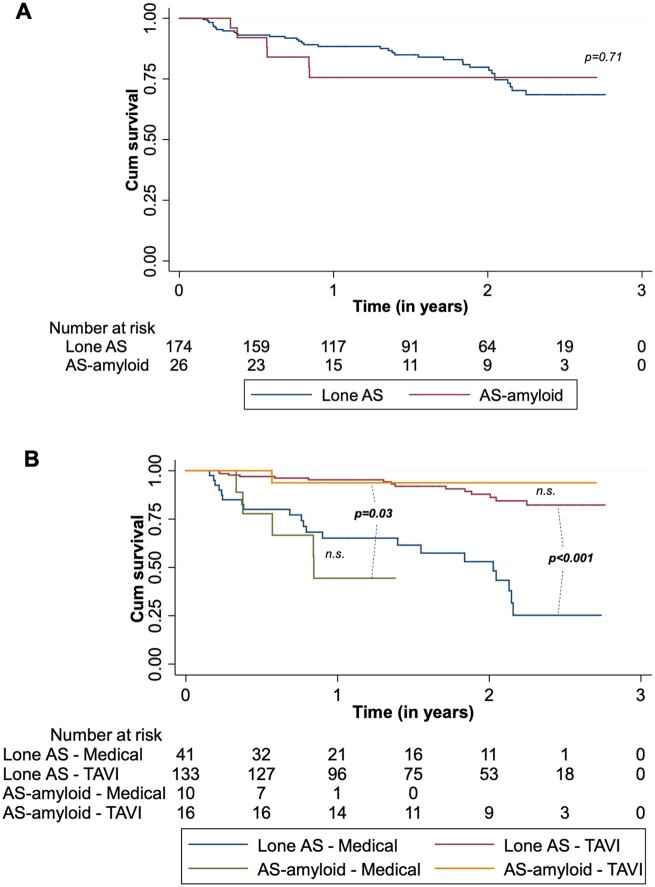
Kaplan–Meier survival curves over a median follow-up of 19 months (interquartile range 10–27 months) by lone AS vs. AS-amyloid (*P* = 0.71) (*A*) and by management strategy (*P* = 0.03 for transcatheter aortic valve implantation vs. medical management in AS-amyloid*, P* < 0.001 for Transcatheter aortic valve implantation vs. medical management in lone AS, *P* = 0.48 in the post-transcatheter aortic valve implantation arm for AS-amyloid vs. lone AS and *P* = 0.39 in the medical management arm for AS-amyloid vs. lone AS) (*B*).

At a median follow-up post-DPD of 19 (10–27) months, 42 (21%) patients had died. AS-amyloid vs. lone AS mortality overall was 6 (23%) and 36 (21%), respectively, *P* = 0.71 (*Figure 3A*), with no difference by DPD Perugini grade, *P* = 0.93 (Supplementary material online, *Figure S4*). Importantly, TAVI improved outcome significantly in the overall cohort (*P* < 0.001) and in AS-amyloid alone (*P* = 0.03) compared to medical management (*Figure 3B*).

**Take home figure:**
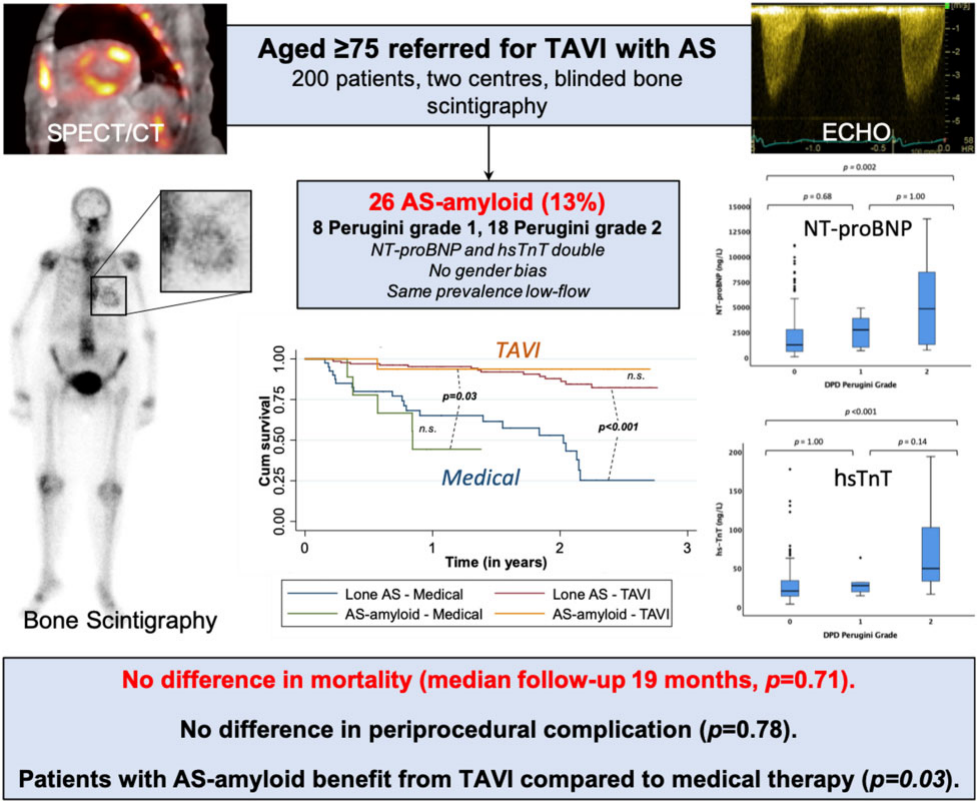
Bone scintigraphy was added to routine clinical care in 200 patients aged 75 and over referred for transcatheter aortic valve implantation with aortic stenosis. The prevalence of dual pathology (AS-amyloid) was confirmed as 13% and was associated with a higher N-terminal pro-brain natriuretic peptide and high-sensitivity troponin T than lone aortic stenosis, however, there was no difference in periprocedural complications or mortality. Furthermore, transcatheter aortic valve implantation significantly improved outcome in patients with AS-amyloid. AS, aortic stenosis; DPD, ^99m^Tc-3,3-diphosphono-1,2-propanodicarboxylic acid; hsTnT, high-sensitivity troponin T; NT-proBNP, N-terminal pro-brain natriuretic peptide; TAVI, transcatheter aortic valve implantation.

## Discussion

These data show that AS-amyloid is common and affects around one in eight elderly patients with severe AS being considered for TAVI. It is different to lone AS with the patients being slightly older, with reduced quality of life (which may, in part, be explained by the age difference^29^) elevated biomarkers, lower voltages, and lower voltage to mass ratio. Strikingly AS-amyloid appears different to lone ATTR-CA with the same prevalence in males and females, and Grade 2 is more common than Grade 1. Findings were also different to prior reports of AS-amyloid: with no difference in disproportionate hypertrophy and no higher prevalence of low-flow aortic stenosis. Periprocedural complications and mortality of AS-amyloid were the same as lone AS. Importantly, TAVI improved outcome in AS-amyloid significantly compared to medical management, disproving the preconception that TAVI may be futile in these patients.

Looking closer, the NT-proBNP and hsTnT elevations were both higher in AS-amyloid suggesting a potential role for these in the diagnostic work-up and surveillance, but ranges were wide. The DPD grade split is striking. This study effectively screened for an occult bystander disease. To discover that the higher burden of the disease (amyloid) is more prevalent than the lower burden (DPD Grade 2 69% vs. Grade 1 31%) does not fit a conventional model of amyloid accumulation and discovery by chance (Grade 1 should be more prevalent). We hypothesize that there is an interaction: the ‘AS-primed’ myocardium being pro the deposition of amyloid. The absence of DPD Grade 3 AS-amyloid in our cohort may suggest censoring by mortality, but this is beyond the scope of this study. Two other observations support interaction: firstly, the discovered prevalence is higher than it is thought to be in healthy ageing and secondly, the lack of survival disadvantage (even though AS-amyloid patients are older) hints that TAVI could be treating the amyloid by reversing pro-amyloidogenic substrate changes. This work highlights our incomplete knowledge about amyloid prevalence in healthy ageing and raises other questions about whether, if AS primes the myocardium for amyloid deposition what other common cardiac conditions do the same? The similar male: female burden could be because AS primes the myocardial interstitium for amyloid regardless of gender, but it could also be that other studies have underestimated female involvement through referral bias and gender dimorphism in the male myocyte hypertrophic response (seen in AS^30^ and Fabry disease^31^) meaning that for the same degree of infiltration, left ventricular hypertrophy is more manifest in males.

Importantly, the presence of dual AS and amyloid pathology was not associated with increased mortality. Transcatheter aortic valve implantation improved outcome significantly in AS-amyloid compared to medical therapy, which is perhaps not unsurprising as it is unloading an already stiff ventricle. Whether this reduction in afterload would be beneficial in moderate AS with ATTR will require further studies—a concept that is being tested in heart failure with moderate AS in the TAVI-unload study (NCT02661451). Furthermore, given that AS-amyloid patients were older, with a worse baseline quality of life and markedly elevated biomarkers, this lack of increased mortality is striking, particularly given the increased mortality seen in patients with such dual pathology following surgical aortic valve replacement.^32^ We propose that there may be a protective effect of the TAVI in AS-amyloid—and that mechanical afterload reduction may actually rebalance amyloid deposition to regression, however, further study is needed. It is clear from this data that teams should not be withholding TAVI in AS-amyloid (wild-type ATTR-CA DPD Grade 1 or 2).

We have alluded to some unanswered questions about AS-amyloid biology, but there are many more. The elevated biomarkers clearly suggest this is disease, but whether some (especially Grade 1) can sometimes be considered bystander is unknown. We do not know if patients receive the same functional benefit to intervention and how biomarkers respond, and we do not know if the amyloid burden changes. There are hints that AS-amyloid is not like ‘typical’ cardiac amyloid so we should not assume that the impact of amyloid-specific therapies such as tafamidis,^33^ patisiran,^34^ and inotersen^35^ will be the same as in lone amyloid. Given the prevalence of AS and the rates of TAVI (which have now exceeded that of surgical aortic valve replacement), we suggest that a study of amyloid therapy in AS-amyloid is urgently needed.

### Limitations

Blinding pre-procedure was broken for two reasons: seven patients had a plasma cell dyscrasia necessitating unblinding as per protocol and some DPD scans were performed clinically (*n* = 16)—we estimate that we would have recruited around half these patients anyway. Not all patients underwent TAVI, particularly in the AS-amyloid group. Follow-up was only mortality, and there was no short-term functional assessment. The study is underpowered to compare DPD Grade 1 to Grade 2.

## Conclusions

AS-amyloid is common and affects around one in eight elderly patients with severe AS being considered for TAVI. It is different to lone AS but has unexpected features that require more investigation and there are suggestions of a biological interaction between the AS and the amyloid. TAVI significantly improved outcome in AS-amyloid, while periprocedural complications and mortality were similar to lone AS, suggesting that TAVI should not be denied to patients with AS-amyloid.

## Funding

This work was funded by a British Heart Foundation Clinical Research Training Fellowship for Dr Paul Scully (FS/16/31/32185).

**Conflict of interest:** P.R.S. is supported by a British Heart Foundation Clinical Research Training Fellowship (FS/16/31/32185). K.P.P. is supported by an unrestricted educational grant from Edwards Lifesciences. T.A.T. is supported by a clinical lecturer grant from the National Institute of Health Research (NIHR, UK). F.P. has received research support from Siemens Healthineers and this work forms part of the translational research portfolio of the NIHR Cardiovascular Biomedical Research Centre at Barts Heart Centre, which is supported and funded by the NIHR. M.M. has received grants and personal fees from Edwards Lifesciences and personal fees from Abbott Vascular. J.C.M. is directly and indirectly supported by the UCLH NIHR Biomedical Research Centre and Biomedical Research Unit at UCLH and Barts, respectively. The remaining authors have no relevant conflict of interest.

## Supplementary Material

ehaa170_Supplementary_Data
