# Supplementary material for: Prevalence and outcome of dual aortic stenosis and cardiac amyloid pathology in patients referred for transcatheter aortic valve implantation
Source: Eur Heart J. 2020 Apr 8;41(29):2759–67. doi: 10.1093/eurheartj/ehaa170 (PMC7395329; doi:10.1093/eurheartj/ehaa170)
Supplement: ehaa170_Supplementary_Data [file eurheartj_41_29_2759_s6.zip › ehaa170-suppl_data/Supplementary Table 3.docx]

| Multivariate Analysis | | | |
| --- | --- | --- | --- |
| Variable | ***p*-value** | **Exp (B)** | **CI for Exp (B)** |
| Age (per year increase) | 0.16 | 1.11 | 0.96-1.29 |
| Gender (male) | 0.12 | 3.18 | 0.74-13.75 |
| V/M Ratio (per mV/g/m^2^ decrease) | **0.045** | 0.00 | 0.00-0.13 |

**Supplementary table 3:** Multivariable binary logistic regression analysis for voltage/mass ratio. Performed separately to avoid underpowering the main model by excluding the 30% of patients with bundle branch block or a ventricular paced rhythm. CI = 95% confidence interval, Exp (B) = exponentiation of the B coefficient, V/M ratio = voltage/mass ratio.
