# Supplementary material for: Prevalence and outcome of dual aortic stenosis and cardiac amyloid pathology in patients referred for transcatheter aortic valve implantation
Source: Eur Heart J. 2020 Apr 8;41(29):2759–67. doi: 10.1093/eurheartj/ehaa170 (PMC7395329; doi:10.1093/eurheartj/ehaa170)
Supplement: ehaa170_Supplementary_Data [file eurheartj_41_29_2759_s6.zip › ehaa170-suppl_data/Supplementary Table 4.docx]

| Complication | AS-amyloid  (n=16) | Lone-AS  (n=133) |
| --- | --- | --- |
| AKI | 1 | 8 |
| Arrhythmia/conduction disturbance | 3 | 16 |
| Bleeding  Minor  Major  Life-threatening | 1  0  0 | 8  1  1 |
| More than mild AR | 1 | 9 |
| Valve stenosis  Mild  Moderate/severe | 1  0 | 0  1 |
| Valve-in-valve | 0 | 2 |
| Vascular  Minor  Major | 0  1 | 4  3 |
| Stroke/TIA | 0 | 6 |

**Supplementary table 4:** a breakdown of post-TAVI complications in the lone-AS and AS-amyloid cohorts, using the Valve Academic Research Consortium-2 (VARC-2) criteria [28]. AKI = acute kidney injury, AR = aortic regurgitation, TIA = transient ischemic attack.
