# Supplementary figures and images for: Prevalence and outcome of dual aortic stenosis and cardiac amyloid pathology in patients referred for transcatheter aortic valve implantation
Source: Eur Heart J. 2020 Apr 8;41(29):2759–67. doi: 10.1093/eurheartj/ehaa170 (PMC7395329; doi:10.1093/eurheartj/ehaa170)

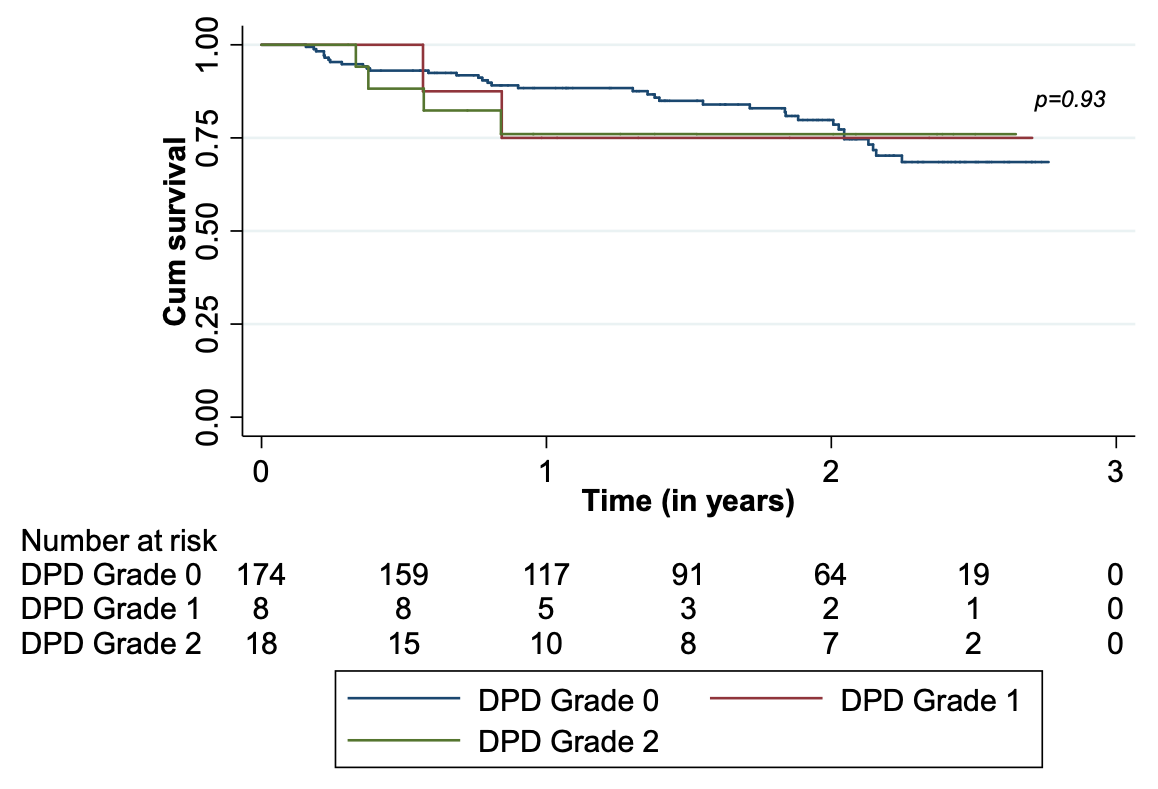

Supplement: ehaa170_Supplementary_Data [file eurheartj_41_29_2759_s6.zip › ehaa170-suppl_data/Supplementary Figure 4.png]
